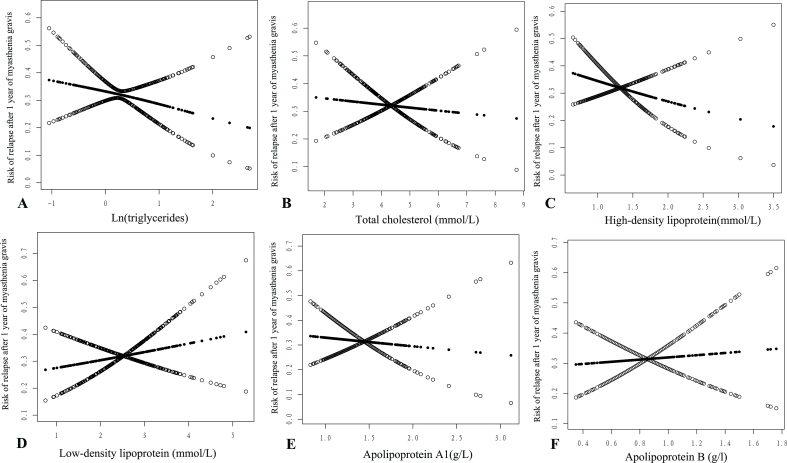# Association between baseline lipid profile and risk of worsening in patients with myasthenia gravis: A retrospective cohort study

**DOI:** 10.1016/j.heliyon.2024.e36737

**Published:** 2024-08-22

**Authors:** Yifan Zhang, Zhiguo Wen, Cong Xia, Meiqiu Chen, Fang Cai, Lan Chu

**Affiliations:** Department of Neurology, Affiliated Hospital of Guizhou Medical University, Guiyang, China

**Keywords:** Myasthenia gravis, Dyslipidemia, Worsening, Non-linear, Association

## Abstract

**Background:**

Dyslipidemia has been implicated in autoimmunity; however, its association with myasthenia gravis (MG) prognosis is unclear. We aimed to investigate the correlation between baseline lipid profiles and risk of MG worsening.

**Methods:**

This 7-year retrospective cohort study conducted at a Chinese hospital included 264 adult patients with MG. Data on baseline lipids, 1-year worsening, and covariates, including demographics, MG characteristics, comorbidities, and treatments were extracted.

**Results:**

Univariate and multivariate logistic regression analyses failed to show a significant association between the risk of 1-year MG worsening and any of the seven blood lipid-related indicators. However, the subsequent non-linear analysis revealed an inflection point in the risk curve of ln[lipoprotein(a)], at 4.06 (58 nmol/L). The lipoprotein(a) levels on the left side of the inflection point presented a positive significant correlation with the risk of MG worsening (relative risk [RR]: 6.06, 95 % confidence interval [CI]: 1.00–38.57), whereas those on the right side of the inflection point demonstrated no significant correlation (RR: 0.86, 95 % CI: 0.55–1.34).

**Conclusions:**

Except for lipoprotein(a) levels being associated with worsening of myasthenia gravis, most lipid parameters were not associated with changes in the clinical course and severity of myasthenia gravis.we observed that lower levels of lipoprotein(a) were associated with a better prognosis in the interval 7–58 nml/L, whereas beyond this interval this was not observed, suggesting dyslipidemia may impact MG prognosis. Further studies are required to validate these findings.

## Graphical abstract

A 7-year retrospective cohort study at a Chinese hospital involving 264 adult patients with myasthenia gravis (MG) found a non-linear correlation between baseline lipid levels and the risk of MG worsening. Although univariate and multivariate analyses did not show a significant association between blood lipid-related indicators (such as triglycerides, total cholesterol, high-density lipoprotein cholesterol, low-density lipoprotein cholesterol, ApoA1, and ApoB) and the risk of MG worsening, non-linear analysis revealed a inflection point in the risk curve of ln[lipoprotein(a)] at 4.06 (58 nmol/L). The range below 58 nmol/L presented a significantly positive correlation, with a 506 % increase in the risk of MG worsening for each unit increase in ln[lipoprotein(a)]. In contrast, the range above 58 nmol/L demonstrated no significant correlation. These findings suggest that dyslipidemia, particularly abnormal lipoprotein(a) levels, may impact the prognosis of MG, and warrant further validation through additional studies.

**Figure:**
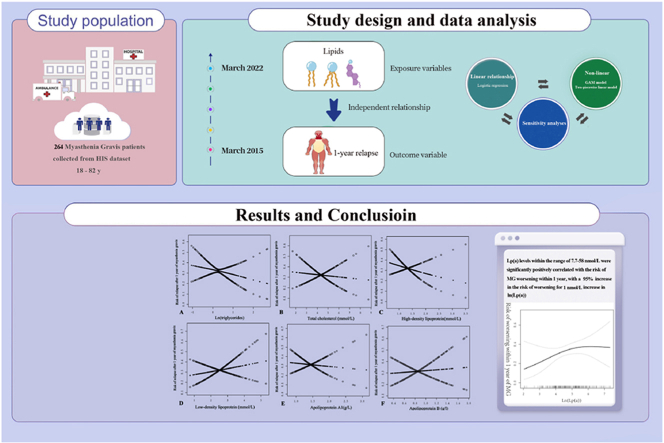

## 1 Background

Myasthenia gravis (MG) is a chronic autoimmune neuromuscular disorder characterized by fatigable weakness affecting the extraocular, bulbar, limb, and axial muscles [1]. Despite its rarity, MG remains an important disease, with a prevalence of 15–179 per 100,000 individuals [2,3]. MG is an antibody-mediated autoimmune disease that disrupts neuromuscular transmission [4]. Its exact etiology remains unclear; however, it is considered to be T cell-dependent and B cell-mediated, with antibodies targeting the neuromuscular junction [5].

Dyslipidemia, a condition characterized by abnormal plasma lipid levels [6], is a known risk factor for cardiovascular diseases [7]. Dyslipidemia has been linked to a variety of diseases, such as diabetes [8], neurodegenerative disorders [9], and autoimmune conditions [[10], [11], [12]]. However, due to insufficient evidence, the specific link between dyslipidemia and MG prognosis remains unclear.

In this study, we propose that dyslipidemia influences the prognosis of MG via several mechanisms. First, dyslipidemia can lead to alterations in the function and quantity of immune cells [[13], [14], [15]], potentially affecting immune system regulation in MG. Second, dyslipidemia may indirectly affect the prognosis of MG by modulating the production and release of inflammatory mediators [16]. Finally, dyslipidemia can disrupt the normal function of immune regulatory factors, potentially affecting the prognosis of MG [17].

To validate these hypotheses, a retrospective cohort study was conducted to explore the correlation between dyslipidemia and MG prognosis. The objective was to deepen our understanding of the influence of dyslipidemia on the long-term outcomes of MG. This study provides valuable insights into the management and treatment strategies for patients with MG with concurrent dyslipidemia.

## 2 Methods

### 2.1 Study population

**Fig. 1:**
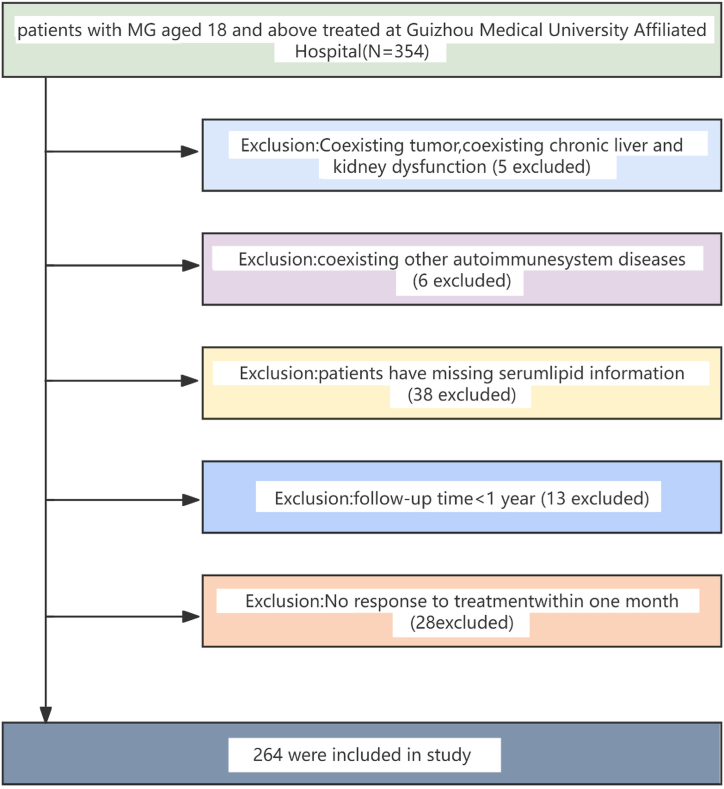
Flowchart of the study process.

This retrospective cohort study was conducted between March 2015 and March 2022 at the Neurology Department of the Guizhou Medical University Affiliated Hospital in Guizhou, China. Patients aged 18 years or older who presented with clinical symptoms and signs consistent with MG were included. No systemic therapy for MG was previously received by any of these patients. In our study, MG was diagnosed in patients who presented with the typical clinical characteristic of MG—fluctuating muscle weakness. An MG diagnosis was established upon meeting any of the following additional criteria: positive pharmacological tests (neostigmine test) and the presence of specific electrophysiological features, such as repetitive nerve stimulation (RNS) tests or the detection of serum anti-acetylcholine receptor (AChR) antibodies, as stipulated in the Chinese guidelines for the diagnosis and treatment of myasthenia gravis (2020 and 2015 editions). Notably, RNS and neostigmine tests are mandatory components of the diagnostic evaluation in our center. Initially, the detection of anti-AChR antibodies was performed in-house and served as an auxiliary diagnostic tool. Despite the later adoption of more sophisticated third-party testing for antibody detection, the reliability of our MG diagnoses is ensured by the consistent application of these neurophysiological and pharmacological examinations. Objective data were continuously and non-selectively collected from the hospital's electronic medical record system. The study included 264 eligible patients, and 264 patients diagnosed with MG were included in the final data analysis after applying the inclusion and exclusion criteria (Fig. 1). Patients who did not meet the diagnostic criteria for MG, had insufficient blood lipid or follow-up information, or had other severe chronic diseases, tumors, or autoimmune systemic diseases were excluded. Furthermore, in our analysis, we have excluded patients with MG who did not exhibit a treatment response within 1 month of initiating therapy. Treatment non-response was defined as follows: for generalized MG, no improvement of more than 3 points in the quantitative myasthenia gravis score (QMGS) and no improvement of more than 2 points in the MG-activities of daily living (ADL) score; and for ocular MG, no improvement of more than 2 points in the ocular QMGS. This study was approved by the Ethics Committee of Guizhou Medical University Affiliated Hospital. Because this was a retrospective study, patient information was anonymized and de-identified, eliminating the need for obtaining informed consent.

### 2.2 Variables

#### 2.2.1 Exposure variable

The exposure variables used in this study were baseline blood lipid levels, including total cholesterol, triglycerides, high-density lipoprotein (HDL) cholesterol, low-density lipoprotein (LDL) cholesterol, apolipoprotein A1 (ApoA1), ApoB and lipoprotein(a)(LP(a)). All tests were conducted at the authors' institution. Individuals who performed blood lipid measurements were not blinded to the patients’ clinical signs, diagnoses, or future research objectives. All lipoprotein (LP)-related data were recorded as continuous variables in a database.

#### 2.2.2 Outcome variable

In this retrospective cohort study, the primary outcome was exacerbation of MG within 1 year of treatment, expressed as a binary variable (Y = 1 for worsening of clinical status and Y = 0 for maintenance of treatment response). MG treatment-response status was assessed 30 days after treatment, and only treatment-responsive patients were included in the follow-up process. The criteria for treatment non-response were established as follows: for generalized MG, lack of improvement exceeding 3 points in the QMGS and lack of improvement exceeding 2 points in the MG-ADL score; for ocular MG, lack of improvement exceeding 2 points in the ocular QMGS. It is important to note that patients receiving cholinesterase inhibitors were required to undergo a minimum 12-h drug withdrawal period prior to QMGS assessments to mitigate any potential medication-related impact on the assessment outcomes. Patients enrolled in the follow-up system had follow-up data collected to monitor any worsening in the previously attained clinical status. Patients were categorized as 1 if they encountered an exacerbation (defined as a >3-point increase in QMGS score or significant worsening of clinical symptoms of MG, as per the Myasthenia Gravis Foundation of America **(**MGFA) definition of MG outcome) within the 1-year follow-up period and 0 if no exacerbation was experienced. Patients lacking clinical data, including the QMGS score, were excluded from the follow-up analysis.

#### 2.2.3 Covariates

The variables were chosen based on clinical experience and the prevailing literature. Demographic information (sex, age) and MG-related characteristics (thymus abnormalities, Osserman classification, MGFA clinical classification, AChR antibodies, and muscle-specific kinase [MuSK] antibodies) were assessed. Initially, from the inception of the study until the year 2018, all samples for AChR and MuSK antibody detection were analyzed using enzyme-linked immunosorbent assay (ELISA). However, starting from 2018, we transitioned to utilizing a third-party testing facility, which implemented radioimmunoassay (RIA) for antibody detection. Extraocular, limb, and respiratory muscles were also included. Various treatment strategies were also considered, including acetylcholine esterase (AChE) inhibitors, steroids, immunosuppressants, immunoglobulins, plasma exchange, rituximab, and thymectomy.

### 2.3 Missing data

Less than 2 % of the data were missing; therefore, multiple imputation methods were not employed to handle missing data (Supplemental Table 1).

### 2.4 Statistical analyses

Continuous variables were reported as mean ± standard deviation for normally distributed variables and as median (minimum value, maximum value) for skewed variables, whereas categorical variables were presented as proportions. Distributions of blood lipids and other covariates were examined in different groups based on worsening status and compared. Independent-samples t-tests were used to compare normally distributed variables, whereas the Mann–Whitney *U* test was used for skewed variables. The chi-squared test was used to compare proportions.

Multivariate logistic regression analysis was performed to assess the independent effects of each exposure variable (lipid-related indicators) on MG worsening, after adjusting for confounders. Effect sizes and 95 % confidence intervals (CIs) are reported. Models with different adjustment levels were constructed to evaluate the stability and reliability of our results. These included unadjusted models (without covariate adjustment), minimally adjusted models (adjusted only for demographic variables), and fully adjusted models (adjusted for all covariates presented in Table 1).

**Table 1:** Baseline characteristics of patients with myasthenia gravis.

Variable	No progression (N = 178)	Progression (N = 86)	P-value
NK cell count, median (min, max), cells/mL	227.50 (53.00–990.00)	160.50 (20.00–726.00)	<0.001^a^
Age, mean ± SD, years	46.31 ± 18.54	42.53 ± 18.28	0.122
TG, Median (min, max), mmol/L	1.74 ± 1.78	1.41 ± 0.91	0.113
TC, mean ± SD, mmol/L	4.40 ± 1.09	4.25 ± 0.96	0.27
HDL-C, mean ± SD, mmol/L	1.34 ± 0.37	1.30 ± 0.37	0.349
LDL-C, mean ± SD, mmol/L	2.52 ± 0.79	2.52 ± 0.77	0.349
ApoA1, mean ± SD, mmol/L	1.47 ± 0.33	1.41 ± 0.31	0.143
ApoB, mean ± SD, mmol/L	0.86 ± 0.27	0.86 ± 0.24	0.99
LP(a), Median (min, max), nmmol/L	4.80 (0.78–7.66)	4.76 (0.66–7.46)	0.234^a^
Sex, n (%)			0.195
Male	56 (31.46 %)	34 (39.53 %)	
Female	122 (68.54 %)	52 (60.47 %)	
Thymus status, n (%)			0.231
Normal	104 (58.43 %)	43 (50.59 %)	
Abnormal	74 (41.57 %)	42 (49.41 %)	
AChR antibody, n (%)			0.036
Negative	80 (44.94 %)	27 (31.40 %)	
Positive	98 (55.06 %)	59 (68.60 %)	
MuSK-ab, n (%)			0.071
Negative	176 (98.88 %)	82 (95.35 %)	
Positive	2 (1.12 %)	4 (4.65 %)	
Osserman classification, n (%)			0.055
Ⅰ	87 (48.88 %)	29 (33.72 %)	
Ⅱ	77 (43.26 %)	46 (53.49 %)	
Ⅲ+IV	14 (7.87 %)	11 (12.79 %)	
MGFA classification, n (%)			0.062
Ⅰ	86 (48.59 %)	29 (33.72 %)	
Ⅱ	35 (19.77 %)	16 (18.60 %)	
Ⅲ	44 (24.86 %)	34 (39.53 %)	
Ⅳ	12 (6.78 %)	7 (8.14 %)	
Involvement of the ocular and extraocular muscles, n (%)			0.205
No	14 (7.91 %)	11 (12.79 %)	
Yes	163 (92.09 %)	75 (87.21 %)	
Involvement of limb muscles, n (%)			0.056
No	113 (63.48 %)	44 (51.16 %	
Yes	65 (36.52 %)	42 (48.84 %)	
Involvement of respiratory muscles, n (%)			0.937
No	163 (91.57 %)	79 (91.86 %)	
Yes	15 (8.43 %)	7 (8.14 %)	
Thymectomy, n (%)			0.169
No	139 (79.43 %)	61 (71.76 %)	
Yes	36 (20.57 %)	24 (28.24 %)	
Use of glucocorticoids, n (%)			0.145
No	43 (24.16 %)	14 (16.28 %)	
Yes	135 (75.84 %)	72 (83.72 %)	
Use of ACh inhibitors, n (%)			0.002
No	45 (25.28 %)	38 (44.19 %)	
Yes	133 (74.72 %)	48 (55.81 %)	
Use of immunosuppressants, n (%)			0.385
No	139 (78.09 %)	63 (73.26 %)	
Yes	39 (21.91 %)	23 (26.74 %)	
Use of intravenous immunoglobulin, n (%)			0.632
No	153 (85.96 %)	72 (83.72 %)	
Yes	25 (14.04 %)	14 (16.28 %)	
Use of plasma exchange, n (%)			0.012
No	178 (100.00 %)	83 (96.51 %)	
Yes	0 (0.00 %)	3 (3.49 %)	
Use of CD20 rituximab, n (%)			0.285
No	166 (93.26 %)	83 (96.51 %)	
Yes	12 (6.74 %)	3 (3.49 %)	

TG, triglycerides; TC, total cholesterol; HDL-C, high-density lipoprotein cholesterol; LDL-C, low-density lipoprotein cholesterol; ApoA1, apolipoprotein A1; ApoB, apolipoprotein B; LP(a), lipoprotein(a); ACh, acetylcholine; AChR, acetylcholine receptor; MuSK, muscle-specific kinase; MGFA, Myasthenia Gravis Foundation of America.

a indicates the use of non-parametric test (Mann–Whitney *U* test).

To address non-linear associations, a generalized additive model was used because binary logistic regression could not handle them. Initially, a penalized spline approach was applied to smooth the association between lipid-related indicators and MG worsening. Subsequently, a recursive algorithm was used to identify the inflection points in the non-linear relationship. At these inflection points, two piecewise linear models were developed to interpret non-linear associations.

All analyses were performed using R software (version 3.6.1; R Foundation, Vienna, Austria) and EmpowerStats (X&Y Solutions, Boston, MA). Statistical significance was defined as a two-tailed P-value <0.05.

### 2.5 Sensitivity analysis

The patient subgroups in this study were administered lipid-lowering medications. However, of the limited patient sample, only six individuals were prescribed lipid-lowering drugs in the year preceding admission. Consequently, owing to its small proportion, we neither adjusted for lipid-lowering drugs nor stratified it as a confounding factor. Conversely, during the sensitivity analysis, patients who were administered lipid-lowering drugs were excluded, and the primary findings were recalculated to assess potential disparities between the two groups.

We also acknowledge that the shift from ELISA to RIA could introduce a measurement bias into the outcome assessment and comparison of results across the different periods of the study. To address this potential source of bias, we conducted a stratified analysis by dividing the study population into two cohorts—those assessed before 2018 and those assessed from 2018 onward. Separate analyses were performed for each cohort to mitigate the impact of differing assay techniques on the study's findings. This stratified approach allowed us to compare the results within each period's framework.

## 3 Results

Table 1 presents a comparison of the baseline characteristics among patients with MG, distinguishing between those who experienced worsening and those who did not. Among the 264 patients studied, 86 had a worsening within 1 year, corresponding to a worsening rate of 32.51 %. Most patients were female (65.91 %). The mean age of the population was 45.08 ± 18.51 years. There were no significant differences (P > 0.05) in the distribution of age; triglycerides; total, HDL, and LDL cholesterol; ApoA1; ApoB; LP(a); sex; thymus status; MuSK antibody; Osserman and MGFA classifications; involvement of respiratory, ocular and extraocular, and limb muscles; thymectomy; and the use of immunosuppressants, intravenous immunoglobulin, or CD20 rituximab therapy between the worsening and non-worsening groups. However, the worsening group showed a higher proportion of AChR antibody positivity and a lower natural killer cell count than did the non-worsening group. Moreover, the percentage of patients in the worsening group who underwent plasma exchange was higher compared to the non-worsening group. Conversely, the worsening group demonstrated lower usage rates of AChE inhibitors than the non-worsening group.

### 3.1 Results of univariate and multivariable logistic regression analysis

Prior to the data analysis, a natural logarithmic function was applied to normalize the skewed distributions of triglycerides and LP(a). Following this predetermined adjustment strategy, the results are shown using three models: an unadjusted model (with no covariate adjustments), a minimally adjusted model (adjusted only for demographic variables), and a fully adjusted model (adjusted for all covariates listed in Table 1). All results are shown in Table 2. The final results revealed no significant associations between triglycerides and MG worsening in the unadjusted model (risk ratio [RR], 0.74; 95 % CI, 0.47–1.17; P = 0.198), after adjusting for sex and age (RR 0.77, 95 % CI 0.48–1.24, P = 0.286), or in the fully adjusted model, which incorporated all predetermined covariates (RR 0.67, 95 % CI 0.34–1.32, P = 0.244. Furthermore, no significant associations were detected for other blood lipid indicators (total, HDL, and LDL cholesterol; ApoA1; ApoB; LP(a); and ln[LP(a)]) in any model. In summary, our findings suggest no significant association between triglycerides, other blood lipid indicators, and the risk of MG worsening, based on the analyzed data.

**Table 2:** Univariate and multivariable analysis results of basal lipid profile associated with 1-year progression in patients with myasthenia gravis.

Exposure	Non-adjusted model RR, 95 % CI, P values	Minimally adjusted model RR, 95 % CI, P values	Fully adjusted model RR, 95 % CI, P values
Ln(TG)	0.74 (0.47, 1.17) 0.198	0.77 (0.48, 1.24) 0.286	0.67 (0.34, 1.32) 0.244
TC	0.87 (0.68, 1.12) 0.270	0.93 (0.71, 1.22) 0.606	0.77 (0.50, 1.19) 0.236
HDL-C	0.71 (0.35, 1.45) 0.348	0.81 (0.38, 1.76) 0.597	0.56 (0.18, 1.71) 0.306
LDL-C	1.01 (0.72, 1.40) 0.971	1.10 (0.77, 1.58) 0.595	1.02 (0.59, 1.76) 0.957
ApoA1	0.53 (0.23, 1.24) 0.144	0.63 (0.25, 1.56) 0.315	0.44 (0.10, 1.92) 0.275
ApoB	1.01 (0.37, 2.74) 0.990	1.39 (0.46, 4.16) 0.557	1.47 (0.29, 7.45) 0.642
Ln (LP(a))	0.90 (0.71, 1.15) 0.397	0.93 (0.73, 1.19) 0.565	1.16 (0.83, 1.62) 0.3883

RR, risk ratio; CI, confidence interval; TC, total cholesterol; HDL-C, high-density lipoprotein cholesterol; LDL-C, low-density lipoprotein cholesterol; ApoA1, apolipoprotein A1; ApoB, apolipoprotein B; LP(a), lipoprotein(a).

Minimally adjusted model is adjusted for sex and age.

Fully adjusted model is adjusted for sex, age, thymus status, acetylcholine receptor antibodies, muscle-specific kinase antibodies, Osserman classification, Myasthenia Gravis Foundation of America classification, natural killer cell count, involvement of limb muscles, involvement of the ocular and extraocular muscles, involvement of respiratory muscles, treatment strategy (use of acetylcholinesterase inhibitors, steroids, immunosuppressants, thymectomy, immunoglobulins, plasmapheresis, CD20 rituximab).

### 3.2 Non-linearity between LP(a) and 1-year MG worsening

**Fig. 2:**
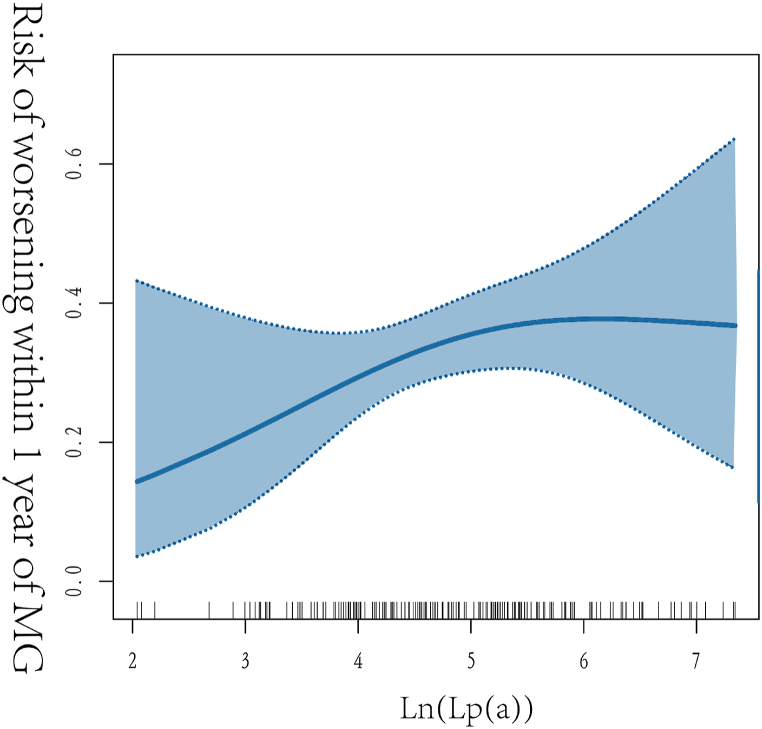
Smooth curve fitting plot. The horizontal axis represents ln(lipoprotein(a)), the vertical axis represents the risk probability of relapse after 1 year, and the open circles represent 95 % confidence intervals.MG, myasthenia gravis; Lp(a), lipoprotein(a).

A curve-fitting analysis was conducted on triglycerides; total, HDL, and LDL cholesterol; ApoA1; ApoB; and LP(a) to assess their relationship with the risk of MG worsening within 1 year. There was a linear relationship between the risk of MG worsening and all indicators (Supplemental Fig. 1) except ln[LP(a)] (Fig. 2). All results are shown in Table 3. The recursive algorithm calculated the inflection point of ln[LP(a)] as 4.06, which was equivalent to 58 nmol/L after applying a natural logarithm transformation. As a result, piecewise linear models were constructed on both sides of the inflection point to explore the relationship between LP(a) and the risk of MG worsening. Significant correlations were observed between the risk of MG worsening and LP(a) levels on the left side of the inflection point (7.7–58 nmol/L). The calculated relative risk was 6.06 (95 % CI, 1.00–38.57, P = 0.05). These findings indicate a positive association between ln[LP(a)] levels and the risk of MG worsening within 1 year. For each unit increase in ln[LP(a)], the risk of MG worsening increased by 506 %; In contrast, no significant correlation was found between ln[LP(a)] and the risk of MG worsening (relative risk: 0.86 [95 % CI, 0.55–1.34], P = 0.503) on the right side of the inflection point (58–1544 nmol/L). In order to enhance the clinical applicability of our findings, we transformed them into the measure of worsening risk per 1-nmol/L increment, yielding the following outcome: in MG patients with a baseline Lp(a) concentration range of 7.7–58 nmol/L, a 1-nmol/L increase in Lp(a) was correlated with a 95 % increased likelihood of worsening in MG within a 1-year period.

**Table 3:** Results of two-piecewise linear model.

Exposure: RR, 95 % CI, P value	Ln(LP(a))
Fitting model using binary logistic regression model	1.16 (0.83, 1.62) 0.3883
Fitting model using two-piecewise linear model	
Inflection point	4.06
< inflection point	6.06 (1.00, 38.57) 0.05
≥ inflection point	0.86 (0.55, 1.34) 0.503
P for log-likely ratio test	0.034

Adjusted for sex, age, thymus status, acetylcholine receptor antibodies, muscle-specific kinase antibodies, Osserman classification, Myasthenia Gravis Foundation of America classification, natural killer cell count, involvement of limb muscles, involvement of the ocular and extraocular muscles, involvement of respiratory muscles, treatment strategy (use of acetylcholinesterase inhibitors, steroids, immunosuppressants, thymectomy, immunoglobulins, plasmapheresis, CD20 rituximab).

### 3.3 Sensitivity analyses

The results were re-evaluated, excluding patients taking lipid-lowering medications. Irrespective of whether they were derived from logistic regression analysis (Supplemental Table 2) or non-linear association analyses (Supplemental Table 3), there was no significant deviation when compared with those procured from the entire population. This finding suggests that the use of lipid-lowering medications did not introduce a bias into our conclusions. Finally, there was no difference upon using the evaluation year as a stratification factor; our results indicate that no significant discrepancies were found between the two cohorts after accounting for the different assays used (Supplemental Table 4).

Furthermore, a sensitivity analysis was conducted to ascertain the influence of varying combinations of diagnostic criteria on the results. In this study, 264 patients with MG were diagnosed based on the presence of a positive neostigmine test result and presence of symptoms. Of the 264 patients, 107 were negative for anti-AChR antibodies, whereas 66 were negative for RNS. Additionally, the results of RNS were negative in 66 patients, and 30 had a double-negative results. Consequently, we conducted a series of analyses, including “symptoms + neostigmine test (+),” “symptom + neostigmine test (+) + anti-AChR antibody (+),” “symptoms + neostigmine test (+) + RNS,” and “symptoms + neostigmine test (+) and exclusion of double-negative patients,” respectively. The data analysis was repeated for the “symptoms + neostigmine test (+) and exclusion of double-negative patients” in four different populations and changes in the results were observed. As illustrated in Supplemental Table 5, despite the reduction in sample size resulting from the change in population, the trend remained unchanged. Specifically, a positive association between LP(a) and risk of MG worsening was observed to the left of the inflection point, as indicated by the two-piecewise linear model.

## 4 Discussion

In this retrospective single-center cohort study, we investigated the correlation between lipid-related parameters and the risk of 1-year worsening in patients with MG. There were no significant associations between the risk of MG worsening and parameters such as triglycerides; total, HDL, and LDL cholesterol; ApoA1; ApoB; and LP(a). However, a non-linear relationship (saturation effect) was detected between LP(a) and the risk of MG worsening. Baseline LP(a) levels in the range of 7–58 nmol/L were positively associated with the risk of MG worsening within 1 year. However, the risk of MG worsening did not increase beyond this interval, even as LP(a) levels continued to rise beyond this interval, showing that this positive association is saturated.

Our findings indicate that lipid abnormalities may play a more nuanced role in the prognosis of autoimmune diseases than previously understood, including in the context of MG. Building on the foundation of previous research, we connected lipid dysregulation with immune dysfunctions that could precipitate or exacerbate autoimmune conditions [[18], [19], [20], [21], [22]]. Increased levels of cholesterol and triglycerides, for instance, have been implicated in activating immune cells and thereby elevating the likelihood of autoimmune reactions [23]. Moreover, dietary factors, such as a high-fat intake, have been experimentally shown to trigger autoimmune thyroid diseases by disrupting immune checkpoints, such as the PD-1 pathway [13].

Clinical parallels have been identified, notably linking HDL cholesterol and ApoA1 status with the prognosis and exacerbation of autoimmune encephalitis [24]. The current study extends this pattern to MG, unveiling a significant positive correlation between LP(a) levels and MG worsening risk. Specifically, we have observed an alarming 506 % increase in the risk of disease worsening for each unit increase in ln[LP(a)] across LP(a) levels of 7.7–58 nmol/L. This pronounced risk elevation underscores the potential impact of dyslipidemia on the pathogenesis and progression of MG.

The mechanistic underpinnings of this correlation may be attributed to the multifaceted roles of lipids in immune regulation. First, LP(a) contains oxidized phospholipids, which can directly activate immune cells such as monocytes and macrophages, promoting the release of inflammatory factors [25]. These inflammatory factors may further activate T cells and B cells, exacerbating the autoimmune response in MG and leading to disease progression [26]. Secondly, LP(a) may increase the level of oxidative stress in endothelial cells and inhibit the synthesis of nitric oxide, leading to endothelial dysfunction. Impaired endothelial function can promote the adhesion and migration of immune cells, exacerbating the local inflammatory response, and consequently affecting the MG condition [27]. Additionally, LP(a) may influence the distribution and clustering of membrane receptors on immune cells by affecting the lipid raft structure on the cell membrane, thereby modulating the signal transduction process in immune cells. This may lead to changes in the activation threshold of immune cells, affecting the maintenance of immune tolerance and exacerbating the autoimmune response [28].

This report highlights the importance of LP(a) levels in the diagnosis and management of neuromuscular diseases, such as MG. Our study found that elevated LP(a) levels, even within the normal range, may be associated with an increased risk of disease progression in MG. This finding expands our understanding of the clinical significance of LP(a) and suggests that it may play a unique role in cardiovascular health as well as in autoimmune disease progression. Thus, consideration of LP(a) levels in routine surveillance, particularly in patients with MG, may facilitate early identification of high-risk individuals and allow for more timely intervention and personalized treatment strategies. Our study underscores the potential of LP(a) as a biomarker in clinical practice and highlights the importance of focusing on subtle changes in LP(a) levels in the context of specific diseases. Therefore, although our findings are based on a single-center cohort and require further validation, this study suggests new perspectives for the treatment of MG. Further, our study provides valuable insight into the development of future therapeutic approaches, possibly driving advances in clinical prediction and intervention strategies.

This study has several strengths. Although this study was retrospective in design, objective data from the hospital's electronic medical record system were used. The data recorders were unaware of the future use of these data in analyzing the relationship between lipids and MG worsening, which mitigated the effects of recall and observational bias on the study results. We also addressed the issue of missing data by first identifying the potential impact of these missing data on the results, and we did not find that these variables introduced bias. In addition, we assessed the extent of the missing data and applied appropriate methods based on our assessment. Although this was an observational study, a rigorous adjustment strategy was implemented to minimize the impact of confounders on the results. We also performed a detailed sensitivity analysis to confirm the robustness of the results.

However, there are some limitations to this study. First, because this is a single-center retrospective observational study, caution should be exercised in generalizing the results. Second, owing to the observational design, we could only establish associations rather than causations. Third, we could only adjust for measurable confounders; unadjusted factors were not considered. Fourth, the possibility of overdiagnosis must be considered, which may have led to the inclusion of some non-MG patients in our study. The MG criteria used in this study were based on the Chinese guidelines for the diagnosis and treatment of myasthenia gravis (2020 and 2015 editions), which include pharmacological tests (neostigmine test), electrophysiological features (RNS tests), and the detection of serum anti-AchR antibodies. Although these criteria are comprehensive and widely accepted, it is important to note that some patients, particularly those who are antibody-negative, may still be diagnosed with MG based on clinical symptoms and response to treatment. This approach ensures that a broad range of MG presentations are captured, although it may introduce some variability in the diagnostic process. Although the inclusion of non-MG patients may increase the proportion of patients who are not expected to deteriorate, these misdiagnosed patients may actually deteriorate owing to the lack of appropriate treatment or other factors (e.g., placebo effect). Thus, the impact of misdiagnosis on the results of our study is unclear and is to be mentioned among the study limitations.

## 5 Conclusion

This study did not find a significant association between blood lipid-related indicators (such as triglycerides; total, HDL, and LDL cholesterol; ApoA1; and ApoB) and the risk of MG worsening within 1 year. However, further non-linear analysis revealed that baseline LP(a) levels in the range of 7.7–58 nmol/L were positively associated with the risk of MG worsening within 1 year. The risk of MG worsening did not increase beyond this interval, even as LP(a) levels continued to rise beyond this interval, showing that this positive association is saturated. These findings suggest that abnormal LP(a) levels may affect the prognosis of MG, providing new insights into the role of lipid metabolism abnormalities in the pathogenesis of MG. This discovery provides a new basis for developing individualized treatment strategies for MG, and future efforts to improve lipid metabolism abnormalities may help reduce the risk of MG worsening. Although this was a single-center retrospective study, the results offer important implications for further exploring the relationship between lipid metabolism and MG prognosis, and warrant validation through additional studies.

## Ethics approval and consent to participate

This study was approved by the Ethics Committee of Guizhou Medical University Affiliated Hospital (Approval Number: 2022-513). Because this was a retrospective study, we anonymized and de-identified patient information, eliminating the need for obtaining informed consent.

## Consent for publication

Not applicable.

## Availability of data and materials

In compliance with legal and ethical obligations, we are unable to share the data used in this study. The data contain potentially sensitive information and are subject to ownership restrictions imposed by third-party organizations. Consequently, public sharing of data is not feasible. We welcome interested researchers to request access to the data by contacting the corresponding author via email, providing relevant background and purpose that meet the criteria for accessing confidential information. We will consider requests based on the appropriate procedures and conditions.

## Competing interests

All authors declare that they have no conflicts of interest.

## Funding

This study was funded by the Guiyang Science and Technology Bureau (NO.[2022]-4-2-8).

## Data availability statement

Data will be made available on request. Interested researchers may contact the corresponding author for data access.

## CRediT authorship contribution statement

**Yifan Zhang:** Writing – review & editing, Writing – original draft, Funding acquisition, Data curation. **Zhiguo Wen:** Investigation, Data curation. **Cong Xia:** Writing – review & editing, Investigation, Data curation. **Meiqiu Chen:** Investigation, Data curation. **Fang Cai:** Investigation, Data curation. **Lan Chu:** Writing – review & editing, Writing – original draft, Methodology, Formal analysis, Conceptualization.

## Declaration of competing interest

The authors declare the following financial interests/personal relationships which may be considered as potential competing interests:YIfan Zhang reports financial support was provided by Guiyang Science and Technology Bureau. If there are other authors, they declare that they have no known competing financial interests or personal relationships that could have appeared to influence the work reported in this paper.

## Figures and Tables

**figs1:**