# Supplementary material for: Association between baseline lipid profile and risk of worsening in patients with myasthenia gravis: A retrospective cohort study
Source: Heliyon. 2024 Aug 22;10(17):e36737. doi: 10.1016/j.heliyon.2024.e36737 (PMC11402134; doi:10.1016/j.heliyon.2024.e36737)
Supplement: Multimedia component 1 [file mmc1.docx]

Supplemental table 1: The missing data description

| Variables | Without missing | Missing | Missing rate (%) |
| --- | --- | --- | --- |
| Age | 260 | 4 | 1.52 |
| TG | 264 | 0 | 0.00 |
| TC | 264 | 0 | 0.00 |
| HDL-C | 264 | 0 | 0.00 |
| LDL-C | 264 | 0 | 0.00 |
| ApoA1 | 264 | 0 | 0.00 |
| ApoB | 264 | 0 | 0.00 |
| Lp(a) | 264 | 0 | 0.00 |
| Sex | 264 | 0 | 0.00 |
| Thymus statu | 260 | 4 | 1.52 |
| AChR-ab） | 264 | 0 | 0 |
| MUSK-ab | 264 | 0 | 0 |
| Osserman classification | 264 | 0 | 0 |
| Involvement of Limb Muscles | 264 | 0 | 0.00 |
| Involvement of Respiratory Muscle | 264 | 0 | 0.00 |
| Involvement of the ocular and extraocular muscles | 264 | 0 | 0.00 |
| Thymectomy | 263 | 1 | 0.38 |
| Use of acetylcholinesterase Inhibitors | 264 | 0 | 0.00 |
| Use of glucocorticoids | 264 | 1 | 0.00 |
| Use of Immunosuppressants | 264 | 0 | 0.00 |
| Use of Intravenous Immunoglobulin | 264 | 0 | 0.00 |
| Use of Plasma Exchange | 264 | 0 | 0.00 |
| Use of CD20 Rituximab | 264 | 0 | 0.00 |
| MGFA | 263 | 1 | 0.38 |
| NK cell count | 259 | 5 | 1.89 |

Supplemental table 2: The Univariate and Multivariable Analysis Results of basal lipid profile Associated with One-Year Progression in Patients with Myasthenia Gravis in the population not taking lipid-lowering medications

| Exposure | Non-adjusted model  RR, 95%CI, p values | Minimally-adjusted model  RR, 95%CI, p values | Fully-adjusted model  RR, 95%CI, p values |
| --- | --- | --- | --- |
| Ln(TG) | 0.76 (0.48, 1.19) 0.2279 | 0.80 (0.49, 1.28) 0.3474 | 0.67 (0.34, 1.32) 0.2435 |
| TC | 0.87 (0.67, 1.12) 0.2788 | 0.95 (0.72, 1.25) 0.7204 | 0.77 (0.50, 1.19) 0.2364 |
| HDL-C | 0.67 (0.32, 1.39) 0.2795 | 0.78 (0.36, 1.71) 0.5419 | 0.56 (0.18, 1.71) 0.3057 |
| LDL-C | 1.01 (0.72, 1.41) 0.9560 | 1.14 (0.79, 1.64) 0.4778 | 1.02 (0.59, 1.76) 0.9573 |
| ApoA1 | 0.52 (0.22, 1.26) 0.1481 | 0.65 (0.26, 1.64) 0.3594 | 0.44 (0.10, 1.92) 0.2753 |
| ApoB | 1.05 (0.38, 2.90) 0.9219 | 1.54 (0.51, 4.68) 0.4479 | 1.47 (0.29, 7.45) 0.6415 |
| Ln (Lp(a)) | 0.95 (0.75, 1.19) 0.6516 | 0.96 (0.76, 1.22) 0.7570 | 1.17 (0.82, 1.68) 0.3901 |

Adjustment strategy is the same as the fully-adjusted model.

Supplemental table 3: The results of two-piecewise linear model in the population not taking lipid-lowering medication

| Exposure: RR, 95%CI, P value | Ln(LP(a)) |
| --- | --- |
| Fitting model using binary logistic regression model | 1.17 (0.82, 1.68) 0.390 |
| Fitting model using two-piecewise linear model |  |
| Inflection point | 4.06 |
| < inflection point | 9.24 (1.12, 76.41) 0.039 |
| ≥ inflection point | 0.83 (0.52, 1.34) 0.448 |
| P for loglikely ratio test | 0.018 |

Adjustment strategy is the same as the fully-adjusted model.

Supplemental table 4: Association of lipid-related indexes with progression of MG within 1 year using time of antibody detection as a stratified variable

| Exposure | POST-2018 (n=196) | Pre-2018 (n=68) |
| --- | --- | --- |
| Ln(TG) | 0.84 (0.35, 2.04) 0.6989 | 0.37 (0.08, 1.75) 0.2097 |
| TC | 1.02 (0.59, 1.76) 0.9375 | 0.48 (0.17, 1.36) 0.1691 |
| HDL-C） | 0.56 (0.14, 2.27) 0.4203 | 0.31 (0.01, 8.71) 0.4912 |
| LDL-C | 1.71 (0.83, 3.56) 0.1481 | 0.50 (0.11, 2.31) 0.3778 |
| ApoA1 | 0.79 (0.13, 4.83) 0.7984 | 0.03 (0.00, 2.02) 0.1000 |
| ApoB | 5.95 (0.68, 52.18) 0.1071 | 0.05 (0.00, 11.82) 0.2847 |
| Ln (Lp(a)) | 1.87 (1.08, 3.25) 0.0260 | 1.28 (0.51, 3.20) 0.5978 |

Adjustment strategy is the same as the fully-adjusted model.

Supplemental table5: Association of lipid-related indexes with progression of MG within 1 year in different diagnostic criteria

| Exposure: RR, 95%CI, P value | neostigmine test +symptoms(n=264) | neostigmine test +symptoms+anti-AchR(n=157) | neostigmine test +symptoms+repetitive electrical stimulation | Exclusion of repeated electrical stimulation and anti-AchR double negative |
| --- | --- | --- | --- | --- |
| Fitting model using binary logistic regression model | 1.16 (0.83, 1.62) 0.3883 | 1.10 (0.74, 1.64) 0.6388 | 1.53 (0.90, 2.62) 0.1153 | 1.18 (0.83, 1.69) 0.3525 |
| Fitting model using two-piecewise linear model |  |  |  |  |
| Inflection point | 4.06 | 4.06 | 4.06 | 4.06 |
| < inflection point | 6.06 (1.00, 38.57) 0.05 | 86.74 (3.93, 1914.75) 0.0047 | 4.94 (0.50, 49.32) 0.1734 | 14.53 (1.52, 138.58) 0.020 |
| ≥ inflection point | 0.86 (0.55, 1.34) 0.503 | 0.51 (0.27, 0.96) 0.0366 | 0.89 (0.59, 2.37) 0.6306 | 0.78 (0.48, 1.26) 0.3081 |
| P for loglikely ratio test | 0.034 | <0.001 | 0.246 | 0.006 |
